# Crosstalk of pyroptosis, ferroptosis, and mitochondrial aldehyde dehydrogenase 2-related mechanisms in sepsis-induced lung injury in a mouse model

**DOI:** 10.1080/21655979.2022.2033381

**Published:** 2022-02-21

**Authors:** Zhenzhen Cao, Hongqian Qin, Yuhui Huang, Yingxue Zhao, Zhipeng Chen, Junfeng Hu, Qin Gao

**Affiliations:** a Department of Respiratory and Critical Care Medicine, The First Affiliated Hospital of Bengbu Medical College, Anhui, P. R. China; b Department of Physiology, Bengbu Medical College, Bengbu, Anhui, P.R. China; c Bengbu Medical College Key Laboratory of Cardiovascular and Cerebrovascular Diseases, Bengbu, Anhui, P.R. China; d Clinical Medical College, Bengbu Medical College, Anhui, P. R. China

**Keywords:** Sepsis-induced lung injury, mitochondrial aldehyde dehydrogenase 2, NOD-like receptor protein-3 inflammasome-mediated pyroptosis, glutathione peroxidase 4, ferroptosis

## Abstract

Acute lung injury (ALI) is a common complication of sepsis. Mitochondrial aldehyde dehydrogenase 2 (ALDH2), an enzyme involved in aldehyde metabolism, exerts a protective effect against sepsis. This study investigated the possible mechanisms underlying the roles of ALDH2, pyroptosis, and ferroptosis in sepsis-induced lung injury. A mouse model of sepsis-induced lung injury was established by cecal ligation and puncture (CLP); lung morphology was evaluated by calculation of lung coefficient, hematoxylin–eosin staining, and electron microscopy. Malondialdehyde (MDA), reactive oxygen species (ROS), and 4-hydroxy-2-nonenal (4-HNE) protein expression levels were used to detect the level of lipid oxidative stress. In addition, total iron was detected using an iron detection kit, and the expression of ferroptosis-related proteins (PTGS2, GPX4), pyroptosis-related proteins, and ALDH2 was examined using western blotting. To further examine the likely mechanisms, the ferroptosis inhibitor ferrostatin 1 (Fer-1), NLRP3 inflammasome inhibitor MCC950, and ALDH2 activator Alda-1 were added. CLP-treated mice exhibited destruction of lung tissue morphology, lipid peroxidation injury, iron content, and increased lung PTGS2 protein expression, accompanied by a decrease in GPX4 protein expression. CLP also downregulated ALDH2 expression and increased the expression of the NLRP3 inflammasome and pyroptosis-related proteins. These adverse effects of CLP were relieved by Alda-1, Fer-1, and MCC950 treatment. In conclusion, both pyroptosis and ferroptosis participate in CLP-induced ALI, and ALDH2 plays a protective role by reducing pyroptosis and ferroptosis. This study provides a scientific basis for the treatment of lung injury in sepsis.

## INTRODUCTION

Sepsis is an infection-induced systemic imbalance inflammatory response that leads to multiple organ dysfunction and remains among the five most common diseases worldwide [1]. Acute lung injury (ALI) is one of the most frequent complications of sepsis because of its pulmonary susceptibility, which occurs the earliest, maintains the highest fatality rate, and progresses rapidly. Excessive inflammation and the aberrant release of reactive oxygen species (ROS) can aggravate damage to pulmonary function in sepsis [2]. However, the precise molecular mechanisms underlying sepsis-induced lung injury remain unclear, and there is an urgent need to develop novel and effective therapeutic strategies.

Different types of programmed cell death, including ferroptosis and pyroptosis, play an important role in maintaining tissue homeostasis and are involved in tissue injury and inflammation-associated diseases [3]. Ferroptosis is triggered by iron-dependent lipid peroxidation caused by redox imbalance, and its morphological characteristics include mitochondrial shrinkage, increase and rupture of membrane density, and decrease or disappearance of mitochondrial cristae [4]. Glutathione peroxidase 4 (GPX4) and prostaglandin endoperoxidase synthase 2 (PTGS2) are biomarkers of ferroptosis, and GPX4, a key regulator of ferroptosis, can reduce toxic phospholipid hydroperoxides into nontoxic phospholipid alcohols, subsequently inhibiting ferroptosis [5,6]. Additionally, excessive intracellular free Fe^2+^ may directly produce excessive ROS through the Fenton reaction, increase oxidative damage, and further promote the production of inflammatory factors such as interleukin-1β (IL-1β) and tumor necrosis factor α as well as toxic lipid peroxidation products, including malondialdehyde (MDA) and 4-hydroxy-2-nonenal (4-HNE), causing severe damage [7,8]. Previous studies have found that excessive ROS levels can promote the activation of the NOD-like receptor family pyrin domain-containing 3 (NLRP3) inflammasome and cause gasdermin D (GSDMD)-mediated pyroptosis, another inflammation-related regulated cell death, exacerbating tissue inflammatory reactions [9]. As inflammation is involved in ferroptosis and pyroptosis, sepsis itself is accompanied with inflammation. Therefore, we wanted to assess whether ferroptosis and pyroptosis are involved in sepsis-induced lung injury and whether they interact with each other.

Mitochondrial acetaldehyde dehydrogenase 2 (ALDH2) is an enzyme expressed in the lungs, heart, brain, and other tissues. We previously found that in diabetes-induced lung injury in rats, ALDH2 can decompose the acetaldehyde metabolite 4-HNE and reduce the oxidative damage of acetaldehyde and its metabolites [10]. It has also been observed in sepsis-related cardiac dysfunction [11]. In addition, studies have shown that activation of ALDH2 inhibits the initiation and activation of NLRP3 inflammasomes by reducing oxidative stress and plays a protective role in atherosclerosis and high glucose-induced myocardial injury [12,13]. In cecal ligation and puncture (CLP)-induced ALI, the expression of pyroptosis-related proteins, such as NLRP3, cysteine-containing aspartate-specific protease 1 (caspase-1), and GSDMD, and the levels of related inflammatory cytokines interleukin-1β (IL-1β) and interleukin-18 (IL-18) were upregulated [14]. In lipopolysaccharide-induced myocardial injury, the downregulation of GPX4 expression increases PTGS2 expression and iron deposition in the myocardium [6,15]. However, only a few studies have assessed whether ALDH2 can play a protective role against sepsis-induced lung injury by inhibiting pyroptosis and ferroptosis.

Therefore, this study investigated the effects of pyroptosis and ferroptosis in CLP-induced lung injury, evaluated whether ALDH2 exerts its protective effects by inhibiting pyroptosis and ferroptosis, and analyzed the possible interaction between pyroptosis and ferroptosis.

## Materials

### Experimental animals

All animal experiments were approved by and conducted in accordance with the Animal Ethics Association of Bengbu Medical College (approval no. 072, 2021). Healthy male specific-pathogen-free (SPF) C57BL/6 J mice (purchased from Skbex Biotechnology Co. Ltd. Certificate No. SYXK (Anhui) 2017–001) were used in the laboratory of Bengbu Medical College. The mice were maintained in a clean environment with sufficient water and food at a temperature of 23–25°C under 12 h light/dark cycle.

### Main reagents

MCC950 (a small-molecule inhibitor of NLRP3 inflammasome; Cat No. HY-12815A, MedChemExpress, USA); ferrostatin-1 (Fer-1, a specific ferroptosis inhibitor), Alda-1 (a selective ALDH2 activator), and dimethyl sulfoxide (DMSO), Cat No. SML0583, SML0462, and D5879, respectively, purchased from Sigma, USA; MDA and Iron kit (Cat No. A0031 and A03921, respectively, Jiancheng Bioengineering Institute, China); dihydroethidium (DHE; Cat No. S0063; Beyotime, China); anti-ALDH2, anti-GPX4, anti-4-HNE, anti-NLRP3, and anti-GSDMD rabbit antibodies (Cat No. ab108306, ab125066, ab46545, and ab219800, respectively, Abcam, UK); anti-PTGS2 rabbit antibody (Cat No. 12282s, Cell Signaling Technology, USA); anti-Caspase-1 rabbit antibody (Cat No. AF5418; Affinity, China); anti-IL-1β and anti-IL-18 goat antibodies (Cat No. CZU0215071 and YR0918081, respectively, R&D, USA); anti-β-actin mouse antibody (Cat No. 5,402,802, Affinity, China); anti-rabbit IgG (Cat No. 71,010,100, Biosharp, China); anti-goat IgG (Cat No. BST15C09A16C60; BOSTER); anti-mouse IgG (Cat No. GAM007, Multisciences, China).

## Methods

### Animal model duplication and grouping

CLP was conducted as previously described [16]. Twelve hours before the operation, the mice were fasted and not allowed to drink, and the operating table was fixed after anesthesia. The hairs from the middle and lower abdomen were removed with a razor, and after sterilization with alcohol, the middle part of the abdomen was cut along the ventral white line for approximately 1 cm, kneaded gently with forceps to find the cecum. the cecum was pulled from the abdominal cavity and ligated 1/3 from the bottom with a 3–0 aseptic silk thread. An 18 g needle was pierced twice, and a small amount of intestinal contents was extruded and the cecum was placed back into the abdominal cavity. After disinfection, the 4–0 mycelial line was sutured in layers and sterilized again.

Sixty-six healthy male SPF C57BL/6 J mice (body weight: 18–24 g) were randomly divided into six groups: sham, CLP, MCC950+ CLP, Fer-1+ CLP, Alda-1+ CLP (n = 12/group), and DMSO+CLP (n = 6). The mice in CLP, MCC950+ CLP, Fer-1+ CLP, Alda-1+ CLP, and DMSO+CLP groups were treated with CLP, intraperitoneally injected MCC950 at 50 mg/kg 3 h before surgery [17], intraperitoneally injected Fer-1 at 5 mg/kg 1 h after surgery [18], intraperitoneally injected Alda-1 at 10 mg/kg 1 h after surgery [19], and intraperitoneally injected 5% DMSO, respectively. The mice in the sham group were subjected to the CLP procedure, but the cecum was not ligated or pierced.

### Measurement of lung coefficient

Twenty-four hours after modeling, the mice were weighed (g) and sacrificed after anesthesia. The lung tissue was removed immediately and the wet lung weight (mg) was recorded. Lung coefficient was calculated as wet lung weight (mg)/body weight (g) × 100% [10].

### Pulmonary morphology observation by hematoxylin-eosin (HE) staining and calculation of injury score

The left lungs of the mice were removed and fixed in 4% paraformaldehyde for 24 h, dehydrated in gradient ethanol, embedded in paraffin, and observed under a light microscope after HE staining. The pulmonary injury Pathology Score system published by the American Thoracic Society was used to evaluate four criteria: alveolar congestion, hemorrhage, infiltration or accumulation of neutrophils in the alveolar or vascular walls, and alveolar wall thickening or hyaline membrane formation. The lung injury score was accumulated as 0 to 4 points (0: no or very light characteristic pathological changes, 1: mild pathological changes, 2: moderate pathological changes, 3: severe pathological changes, and 4: very severe pathological changes). A cumulative score of lung injury was also obtained [20].

### Pulmonary ultrastructural observation by transmission electron microscopy

The fresh right lungs of mice were prefixed with 2.5% glutaraldehyde at 4°C for 24 h, fixed with 1% osmium acid for 2 h, dehydrated with ethanol, soaked with epoxy, embedded, sectioned, and photographed using transmission electron microscopy (TEM) (Cat No. JEM1400, Japanese electron) [8,21].

### Measurement of lung MDA and tissue iron levels

The thiobarbituric acid (TBA) method was used for detecting MDA in accordance with the instructions of the Nanjing Jiancheng Kit. MDA was condensed with TBA to form a red product in tissues, which produced a maximum absorption peak at 532 nm [15]. Tissue iron was condensed with dipyridyl to form a pink complex, and the sample was observed at a wavelength of 520 nm [7].

### Detection of ROS in the lung tissue of mice using a DHE fluorescence probe

The fresh left-lung tissues of mice were fixed in 4% paraformaldehyde for 24 h, dehydrated with 20%–30% sucrose to the bottom, and embedded in OCT sections. The specimens were incubated at 37°C for 30 min in the dark with 10 μmol/L DHE. After washing with PBS thrice, an anti-fluorescence quenching agent was added to seal the slices. Changes in red fluorescence in the slices were observed and recorded using a Zeiss automatic inverted fluorescence microscope (Cat No. Observer Z1, Germany) in a dark room [15].

### Measurement of expression of related proteins by western blotting

A lung tissue homogenate was prepared using the remaining lungs, the protein concentration was determined using the BCA method, and the same volume of sample was denatured by heating and subjected to SDS-PAGE after cooling, following which the protein was transferred to a PVDF membrane by a wet transfer method after approximately 60 min. ALDH2 (1:3000), GPX4 (1:3000), PTGS2 (1:1000), 4-HNE (1:3000), NLRP3 (1:1000), Caspase-1 (1:2000), GSDMD (1:1000), IL-1β (1:2000), IL-18 (1:2000), and β-actin (1:3000) were added and the sample was refrigerated at 4°C overnight. The next day, the gel was washed with Tris Buffer Solution Tween (TBST) buffer on the shaker thrice. Anti-rabbit (1:8,000), anti-goat (1:8,000), and anti-rat (1:8,000) related secondary antibodies were prepared and applied, followed by incubation at room temperature(25°C) for 1 h. The gel was then washed with TBST buffer on the shaker four times. The exposure solution was applied to the PVDF membrane, the gel was exposed to a gel imager, and observations were recorded [13,14].

### Statistical analysis

The results were analyzed using the SPSS software (version 26.0). The mean ± SD was used to express the data, and one-way analysis of variance was used to measure the data; the difference was statistically significant at P < 0.05.

## Results

The present study explored changes in pyroptosis and ferroptosis in sepsis-induced ALI and analyzed the effect of ALDH2 and its underlying mechanism of action. We found that ALDH2 improved lung tissue morphology and alleviated lipid peroxidation. These effects of ALDH2 may be related to the inhibition of pyroptosis and ferroptosis. More importantly, these three factors may interact with each other.

### Changes in mouse lung coefficient in each group

The lung coefficient (lung weight/body weight) reflects lung tissue inflammation and edema. With an increase in the inflammatory response, the lung tissue weight and coefficient increased correspondingly. As shown in Table 1, compared with that in the sham group, the lung coefficient in the CLP group was higher, whereas when compared with those in the CLP group, the lung coefficients in the MCC950+ CLP, Fer-1+ CLP, and Alda-1+ CLP groups were correspondingly lower. No change was observed in the DMSO+CLP group as compared to that in the CLP group. There were no differences between the MCC950+ CLP, Fer-1+ CLP, and Alda-1+ CLP groups. These results suggest that lung inflammation and swelling occur in CLP mice and that lung injury can be improved by inhibiting pyroptosis and ferroptosis and activating ALDH2.

**Table 1.** Comparison of mouse lung coefficients in each group (mean ± SD, n = 6)

Groups	Weight	Lung wet weight	Lung coefficient
Sham	20.20 ± 1.18	104.17 ± 4.83**	5.16 ± 0.14
CLP	21.68 ± 1.78	120.33 ± 5.99*	5.57 ± 0.28*
DMSO+CLP	20.87 ± 1.59	115.50 ± 7.94	5.55 ± 0.13
MCC950+ CLP	21.00 ± 0.71	110.33 ± 4.27^#^	5.25 ± 0.11**^#^**
Fer-1+ CLP	20.07 ± 1.23	106.67 ± 6.21^##^	5.32 ± 0.20^#^
Alda-1+ CLP	21.96 ± 0.97	114.00 ± 8.28^#^	5.18 ± 0.19^#^

*P < 0.05, **P < 0.01 vs Sham group; ^#^P < 0.05, ^##^P < 0.01 vs CLP group

### Changes in HE staining and injury score in mouse lung tissues in each group

**Figure 1.**
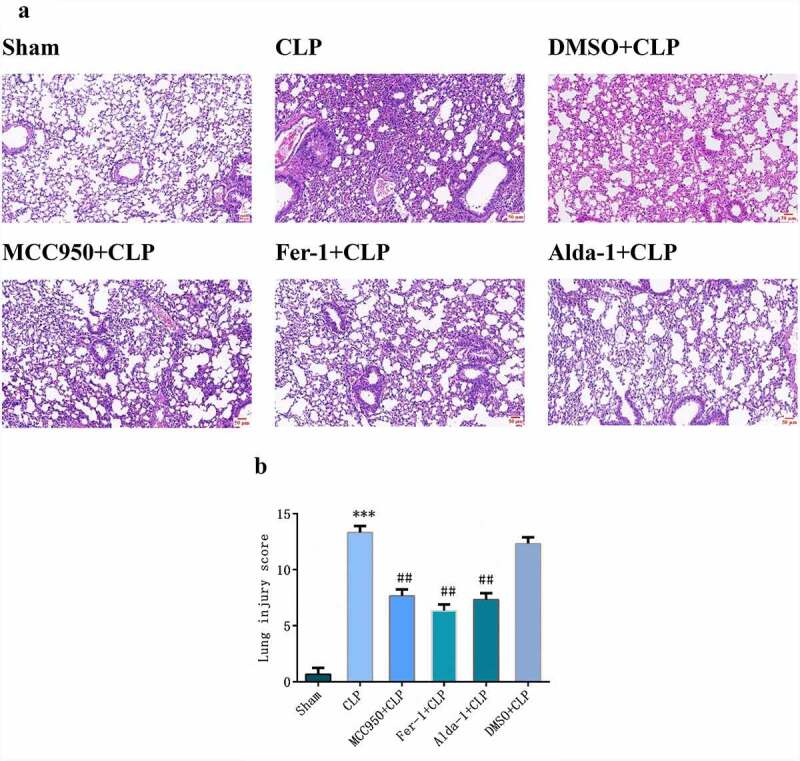
Morphological changes after HE staining (a) (200X, Scale bar = 50 μm) and injury scores (b) in mouse lung tissue in different groups (mean ± SD, n = 3), ***P < 0.001 vs sham group; ^##^P < 0.01 vs CLP group.

As shown in Figure 1, in the sham group, the alveolar structure was complete and there was no neutrophil infiltration, hemorrhage, or edema in the alveolar interstitium. In the CLP group, the alveolar structure was severely damaged, with hyperemia and bleeding, inflammatory cell infiltration, alveolar wall thickening, and increased lung score (P < 0.001). Compared with the CLP group, the MCC950+ CLP, Fer-1+ CLP, and Alda-1+ CLP groups showed less alveolar structure damage, bleeding, congestion, and neutrophil infiltration and decreased lung scores (P < 0.01). In the DMSO+CLP group, there was no significant difference compared to that in the CLP group.

### Ultrastructural changes in lung tissues in each group

**Figure 2.**
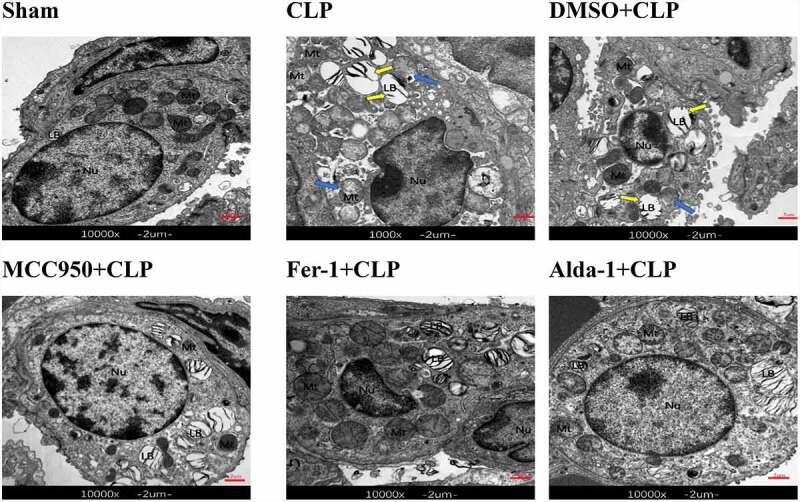
The transmission electron microscopy images of mouse lung tissue (10000x, Scale bar = 2 μm), nucleus (Nu), lamellar bodies (LBs), and mitochondria (Mt).

As shown in Figure 2, the mitochondrial structure in the sham group was clear, and the lamellae were neatly arranged in the lamellar bodies. Compared with that in the sham group, there was increased mitochondrial membrane density, decreased or disappeared mitochondrial cristae, vacuolated lamellar bodies, and shrunken cell nuclei in the CLP group, indicating the presence of ferroptosis. Compared with that in the CLP group, in the MCC950+ CLP, Fer-1+ CLP, and Alda-1+ CLP groups, the destruction of lamellar bodies decreased with reduced vacuolation and mitochondrial structure, the crista was ameliorated, and some mitochondria appeared morphologically normal. There was no significant difference between the DMSO+CLP and CLP groups. Combined with the lung coefficient, HE staining, and TEM results, experimental errors caused by DMSO could be excluded. Therefore, the DMSO+CLP group was not included in the latter experiment.

Blue arrow: mitochondrial disruption, loss, or reduction of the mitochondrial crista. Yellow arrow: vacuolated LBs.

### Changes in MDA and iron levels in lung tissues in each group

**Figure 3.**
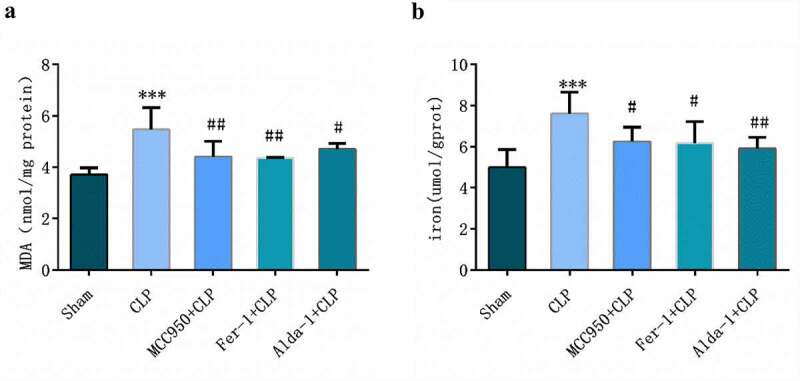
Detection of MDA (a) and iron (b) in lung tissue of mice (mean ± SD, n = 5), ***P < 0.001 vs sham group; ^#^P < 0.05, ^##^P < 0.01 vs CLP group.

As shown in Figure 3, compared with those in the sham group, MDA and tissue iron contents in the CLP group were higher (P < 0.01). However, compared with those in the CLP group, MDA and tissue iron contents in MCC950+ CLP, Fer-1+ CLP, and Alda-1+ CLP groups were lower (P < 0.05–0.001).

### Changes in ROS in lung mouse tissues in each group

**Figure 4.**
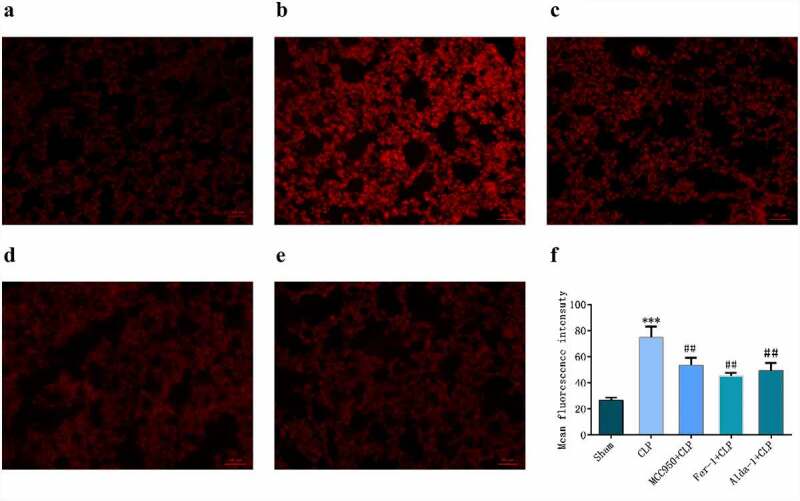
DHE detection of ROS expression and fluorescence statistics in mouse lung tissue in different groups (200X, Scale bar = 50 μm) a: sham group; b: CLP group; c: MCC950+ CLP group; d: Fer-1+ CLP group; e: Alda-1+ CLP group; f: Fluorescent statistical results (mean ± SD, n = 5), ***P < 0.001 vs sham group; ^##^P < 0.01 vs CLP group.

DHE is a common fluorescent probe used for the detecting intracellular superoxide anion levels. When cells were ingested with DHE, the intracellular superoxide anion reacted with biochemical reaction, and when the excitation wavelength was 300–600 nm, red fluorescence was produced, indicating an increase in ROS. As shown in Figure 4, the fluorescence intensity in the CLP group was significantly higher than that in the sham group. In the MCC950+ CLP, Fer-1+ CLP, and Alda-1+ CLP groups, the fluorescence intensity was lower than that in the CLP group.

### Changes in the expression of related proteins in each group

**Figure 5.**
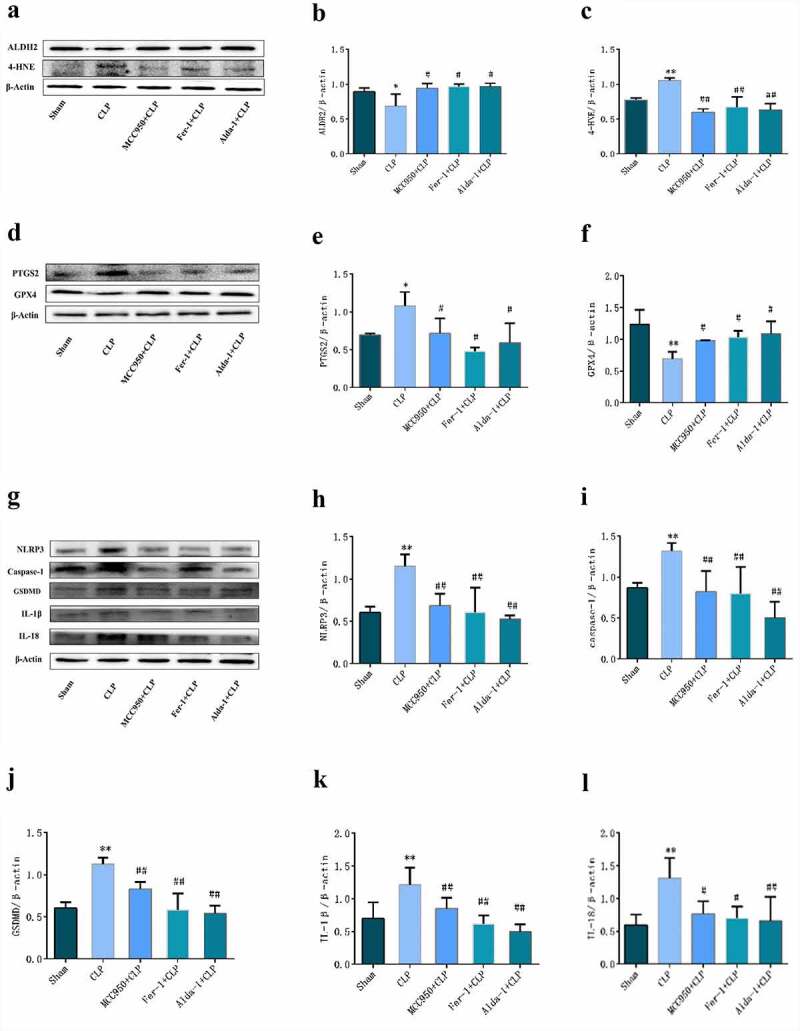
The typical Western blot pictures and statistical results of each group. a, d, g: typical Western blot bands of lung tissue; b, c, e, f, h, i, j, k, l: changes in the expression of different proteins in the lung tissues of each group. (mean ± SD, n = 3), *P < 0.05, **P < 0.01 vs sham group; ^#^P < 0.05, ^##^P < 0.01 vs CLP group.

The results of Western blotting are shown in Figure 5. When compared with that in the sham group, the protein expression of ALDH2 and GPX4 in the CLP group was significantly lower, while the protein expression of PTGS2, 4-HNE, NLRP3, Caspase-1, GSDMD, IL-1β, and IL-18 was significantly increased. When compared with that in the CLP group, ALDH2 and GPX4 protein expression was higher in the MCC950+ CLP, Fer-1+ CLP, and Alda-1+ CLP groups, whereas PTGS2, 4-HNE, NLRP3, Caspase-1, GSDMD, IL-1β, and IL-18 protein expression was significantly lower.

## Discussion

Sepsis is a life-threatening condition caused by an excessive immune response to infection [22]. As ALDH2 protects cells from acetaldehyde and fatty acid-derived aldehydes (such as 4-HNE), we hypothesized that ALDH2 activation is an effective treatment for sepsis-induced lung injury. We established a CLP-induced lung injury model to confirm the role of ALDH2 in ALI and investigate its molecular mechanisms of action. The CLP model shows symptoms similar to those in clinical sepsis patients and is the gold standard model for sepsis [16]. We observed that the lung coefficient in CLP mice increased 24 h after CLP, and HE staining and TEM results showed that the lung structure and lamellar body injury were aggravated, indicating that the sepsis-induced lung injury model was successful. In addition, the levels of MDA and ROS in lung tissue were increased, 4-HNE protein expression was increased, and the release of inflammatory cytokines IL-1β and IL-18 was also increased, which is consistent with previous reports [6,23]. This suggests that lipid oxidative stress and inflammation reaction injury were aggravated in sepsis-induced lung injury.

Excessive ROS levels can promote activation of the NLRP3 inflammasome, causing pyroptosis [24]. In addition, increased inflammatory cell infiltration can promote the synthesis and accumulation of ROS in lung tissues [25,26]. As ROS accumulate in lung tissues, it is possible that different types of cell death occur during ALI. During the development of ferroptosis, iron overload, ROS, and lipid peroxidation accumulation aggravate the occurrence of cell ferroptosis. Additionally, when GPX4, a key enzyme for the removal of lipid ROS, loses its activity, lipid peroxidation is aggravated and causes ferroptosis [27]. PTGS2 is a peroxidase and its increase can be used as a biomarker for ferroptosis [28]. Therefore, in the CLP model, we observed an association between an inflammatory response, oxidative stress injury, and iron content. Additionally, lung GPX4 protein expression was decreased, accompanied by an increase in PTGS2 protein expression. Simultaneously, mitochondria shrank and the cristae disappeared. These results suggested that ferroptosis is involved in CLP-induced lung injury. Previous studies have illustrated that in a rat model, LPS treatment promoted caspase-1 immunoreactivity in astrocytes and caused an increase in the release of pro-inflammatory cytokines, such as IL-1β and IL-18, resulting in neuronal injury [29]. In our study, we observed that the expression levels of the key inflammasome proteins NLRP3 and caspase-1 and pyroptosis key proteins GSDMD, IL-1β, and IL-18 were significantly increased, suggesting that pyroptosis was also involved in CLP-induced lung injury.

Recent studies have reported that pyroptosis and ferroptosis are the main pathogenic mechanisms of sepsis-induced lung injury [13,30]. To further verify the roles of ferroptosis and pyroptosis in sepsis-induced lung injury, we used Fer-1 and MCC950 to inhibit ferroptosis and pyroptosis, respectively. Studies have shown that Fer-1 can alleviate LPS-induced ALI by regulating ferroptosis [7] and pretreatment with Fer-1 reversed the effect of IL-6 on lipid peroxidation and ferroptosis in BEAS-2B cells, while Fe augmented the effect [31]. Meanwhile, Fer-1 also protects HT-22 cells from ferroptosis by decreasing PTGS2 expression and increasing GPX4 protein expression [32]. In our study, we observed that after intraperitoneal injection of Fer-1 in CLP mice, lung morphology injury was alleviated and damage to lamellar bodies and mitochondria was reduced. In addition, MDA and ROS levels decreased, 4-HNE and PTGS2 protein expression decreased, and GPX4 protein expression increased. These results suggest that the inhibition of ferroptosis could reduce oxidative stress and lung damage induced by sepsis. MCC950 is a recently developed small-molecule compound that selectively inhibits the activation of the NLRP3 inflammasome *in vitro* and *in vivo* and inhibits pyroptosis [17]. We observed that when CLP mice were treated with MCC950, lung tissue injury was alleviated; MDA expression and ROS release were decreased. The expression of key inflammasome proteins NLRP3 and caspase-1 and pyroptosis-related proteins GSDMD, IL-1β, and IL-18 was decreased, suggesting that inhibition of pyroptosis also alleviated lung injury in mice with sepsis. These results demonstrate that inhibition of both ferroptosis and pyroptosis could protect against sepsis-induced lung injury. However, identifying the dominant type of cell death and assessing whether there is a sequential relationship between the two different types of cell death needs further study.

Alda-1 is a small-molecule agonist of mitochondrial ALDH2 that can prevent the production of cytotoxic aldehydes [33]. Our previous studies have demonstrated that activation of ALDH2 could reduce the release of 4-HNE, inhibit oxidative stress, alleviate cardiac and lung injury in diabetic rats, and alleviated kidney injury in rats with sepsis [10,13,34]. In this study, we observed a decrease in lung ALDH2 protein expression in mice with sepsis, suggesting that mitochondrial ALDH2 may be an important cause of lung injury in mice with sepsis. After ALDH2 was activated with Alda-1, with an increase in ALDH2 protein expression, lung injury was alleviated, MDA and ROS levels and 4-HNE protein expression were decreased, tissue iron content and PTGS2 protein expression were also decreased, and GPX4 protein expression was increased, suggesting that activation of ALDH2 inhibited ferroptosis. The expression of NLRP3, Caspase-1, GSDMD, IL-1β, and IL-18 was decreased, suggesting that ALDH2 also inhibited pyroptosis in sepsis-induced lung injury.

It is worth noting that when CLP mice were treated with MCC950 to inhibit NLRP3 and pyroptosis and treated with Fer-1 to inhibit ferroptosis, ALDH2 protein expression was also increased, suggesting that ferroptosis and pyroptosis also regulate ALDH2 expression. When CLP mice were treated with MCC950, besides the decrease in NLRP3 and pyroptosis protein expression, GPX4 protein expression was also increased, accompanied by decreases in PTGS2 and 4-HNE protein expression, suggesting that inhibition of pyroptosis also inhibited ferroptosis. However, when CLP mice were treated with Fer-1, in addition to the increase in GPX4 protein expression and the decrease in PTGS2, NLRP3 and pyroptosis protein expression was also decreased, suggesting that inhibition of ferroptosis also inhibited pyroptosis. Ferroptosis and pyroptosis were found to interact in sepsis-induced lung injury in a mouse model. However, the specific mechanism remains to be further explored.

## Conclusions

In summary, our study suggests that pyroptosis and ferroptosis are involved in CLP-induced lung injury in mice. The inhibition of pyroptosis, inhibition of ferroptosis, and activation of mitochondrial ALDH2 play a protective role by inhibiting pyroptosis and ferroptosis. Interactions among ALDH2, pyroptosis, and ferroptosis may also occur.

## Highlights

- Lung pyroptosis and ferroptosis are activated in mice treated with CLP.
- ALDH2 reduces sepsis-induced lung injury by inhibiting pyroptosis and ferroptosis.
- ALDH2, pyroptosis, and ferroptosis interact during sepsis-induced lung injury.
